# Impact of cranial collimation on cephalometric landmark visibility

**DOI:** 10.1186/s13005-026-00586-1

**Published:** 2026-02-05

**Authors:** Kathrin Becker, Sarah Azimi, Manuel Nienkemper, Robert Stigler, Lisa Josefine Langer, Katharina Mücke

**Affiliations:** 1 Department of Orthodontics and Dentofacial Orthopedics, Charité – Universitätsmedizin Berlin, Aßmannshauser Straße 4-6, Berlin, 14197 Germany; 2 Department of Orthodontics, Universitätsklinikum Düsseldorf, Moorenstraße 5, Düsseldorf, 40225 Germany; 3 Department of Orthodontics, Universitätsmedizin Mainz, Johannes-Gutenberg University of Mainz, Augustusplatz 2, Mainz, 55131 Germany; 4 Private Practice, Kasernenstraße 1, Düsseldorf, 40213 Germany; 5 Private Practice, Rainerstraße 2, Vöcklamarkt, 4870 Austria

**Keywords:** Growth pattern, Orthodontics, Radiation protection

## Abstract

**Introduction:**

To reduce radiation exposure, collimation of lateral cephalograms is frequently provided by manufacturers. However, cranial collimation geometries can lead to masking of anatomical reference points that are necessary for cephalometric analyses. To avoid unintended reference point masking, identifying optimal collimation geometries is crucial for diagnostic reliability and is therefore of significant clinical relevance. The aim of this study was to quantify (i) the frequency of Nasion and Sella landmark masking for a common collimation geometry and (ii) to derive ideal collimation parameters.

**Materials and methods:**

*N* = 1000 randomly selected lateral cephalograms were categorized into collimated (*n* = 49) and non-collimated (*n* = 951) images. The non-collimated images were virtually collimated using the same geometric settings. For each landmark and cephalogram, the frequency of landmark masking was assessed. For the statistical analysis log-regression was used to analyze association with head positioning, i.e. angular discrepancy to Frankfort horizontal plane (FHP-discrepancy), and Bjork sum growth pattern. The analyses were performed using the open-source software R using a significance level alpha of 0.05.

**Results:**

*Sella* was visible on all radiographs, whereas *Nasion* was masked in 0.6% of the lateral cephalograms. Masking could have been avoided if collimation was limited to the upper 16.02% of image height. Counter clockwise inclination of the head measured as FHP-discrepancy was significantly associated with landmark masking (*p* = 0.040) as well as a vertical skeletal growth pattern (as determined by the Bjork sum) (*p* = 0.029).

**Conclusions:**

The evaluated collimation geometry that masks the upper 18% of the radiographs would have prevented masking for 99% of the included radiographs. Correct head positioning might further reduce the risk of landmark masking. The benefit of cranial collimation should be considered carefully for patients with pronounced vertical growth pattern (as determined by the Bjork sum).

**Supplementary Information:**

The online version contains supplementary material available at 10.1186/s13005-026-00586-1.

## Introduction

Orthodontic treatment planning involves diagnostical radiological exams. Commonly, an orthopantomogram is taken and frequently, also a lateral cephalogram is recorded [1]. The latter allows for assessment of skeletal and dental relationships, deviations from the norm as well as estimation of the patient specific growth pattern [2, 3]. For this purpose, landmarks are identified on the lateral cephalograms and connected by lines to create angles.

The European Guidelines on Radiation Protection in Dental Radiology recommend to reduce radiation exposure as much as reasonable achievable and clinically justifiable [4]. In spite of today’s advanced digital imaging, clinicians are obliged to minimize radiation exposure as much as possible according to the ALARA principle (“As Low As Reasonably Achievable”) or the ALADA-IP principle (“As low as diagnostically acceptable being indication-oriented and patient-specific”) [5].

Techniques to reduce radiation dose include collimation, filtering of the spectrum of the x-ray beam and usage of lead aprons [6, 7].

For cephalometric analyses, it is required that, with or without collimation, all relevant anatomical landmarks are visible on the radiograph. Therefore, optimal collimation geometries are crucial. Clinical practice however indicated that *Nasion* masking may occur in some patients when applying cranial collimation in lateral cephalograms, thus posing the need for geometries that prevent the necessity of capturing a second radiograph.

The aims of the present study were (i) to quantify the risk of landmark masking for a common collimation geometry, (ii) to assess correlation of landmark masking with head positioning and growth pattern, and (iii) to derive recommendation for ideal collimation geometries As currently, there is a lack of evidence to what extent cranial collimation is associated with an increased risk of landmark masking, and whether landmark masking is associated with malpositioning of patients during the radiological examination or growth pattern, this study holds significant clinical importance.

## Materials and methods

### Study design

A total of *n* = 1000 anonymized lateral cephalograms from patient records of the Department for Orthodontics, University Hospital Dusseldorf, Germany were included in the study. The images were taken with the Orthophos XG and Orthophos SL 3D (Sirona Dental Systems GmbH, Bensheim, Germany). All patients were positioned similarly following a standardized instruction where the patients were aligned according to the Frankfort Horizontal plane with the help of ear rods and a forehead rest.

The study protocol was approved by local ethical committee from Heinrich-Heine University of Dusseldorf, Germany (ref. no. 2021 − 1750).

The number *n* = 1000 was selected a priori in order to have a sufficient sample size for statistical evaluation. However, since no likewise study could be identified in literature, no sample siye calculation was possible.

**Fig. 1:**
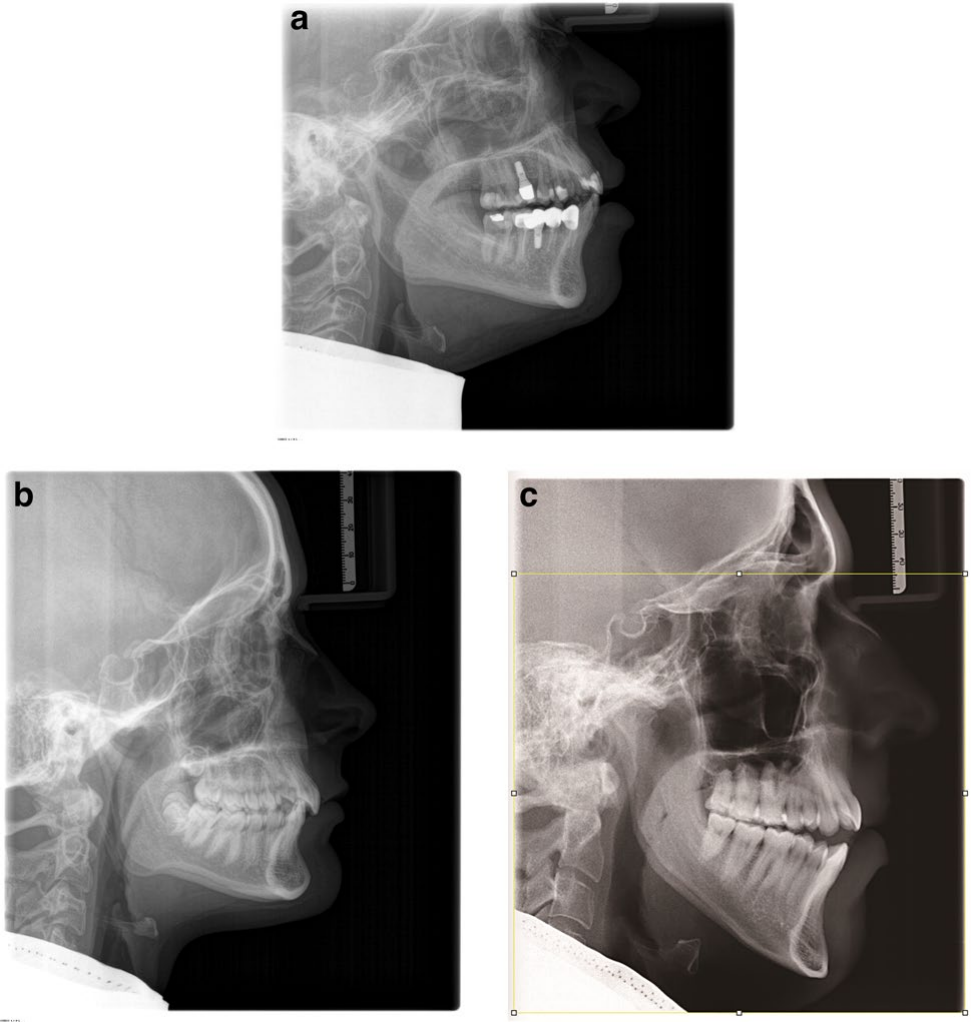
**a** Lateral cephalogram with cranial collimation of Nasion. **b** Lateral cephalogram without cranial collimation. **c **Lateral cephalogram with virtual cranial collimation of Nasion

### Eligibility criteria

Both cranially collimated (Fig. 1a) as well as non-collimated lateral cephalograms (Fig. 1b) were included for the present investigation. Individual demographic details (e.g. sex distribution, age or clinical indication) were not collected for the present investigation.

The distribution of cranially collimated and non-collimated lateral cephalograms reflects routine imaging of the institution, where cranial collimation is selected for patients on an individual basis since standardized criteria for the application of cranial collimation are lacking. Furthermore, images with significant motion or metallic artifacts that could interfere with landmark visibility were not included in the study.

### Radiological image classification

The lateral cephalograms were classified into cranially collimated (*n* = 49) and non-collimated (*n* = 951) images.

The non-collimated images were virtually collimated (Fig. 1c) by applying the geometry provided by the manufacturer (Sirona Dental Systems GmbH, Bensheim, Germany), in which the upper 18% of the image height are collimated. This was done by using the open-source programme Fiji for image processing [8]. 

All images were then labelled as follows:

- Label I: all relevant landmarks remained visible after collimation.
- Label II: not all relevant landmarks for the diagnosis remained visible after collimation.

Furthermore, the frequency of non-masking of *Nasion* and *Sella* was assessed and the optimal percentage for collimation, i.e. at which all landmarks would have remained visible, was computed based on the coordinates of these two landmarks.

### Cephalometric analysis

To determine the skeletal growth pattern and the anteroposterior skeletal disharmony, the Bjork sum according to the Bjork-Jarabak analysis [9] and the Frankfort-mandibular-plane-angle [10, 11] were assessed by using the artificial intelligence (A.I.) driven web-based orthodontic diagnostic software “WEBCEPH” (AssembleCircle Corp., Republic of Korea). The landmarks were automatically placed by the AI and manually verified by a trained observer (SA). The cephalometric landmarks, angles and lines that were used for the measurements are provided in Table 1, 2 and 3. They were calculated using the Webceph software to examine the patient specific growth patterns and manually corrected when needed. In two of the collimated lateral cephalograms, in which Nasion was masked, its position was estimated by a trained observer (SA) approximately guessing where the most anterior point of the frontonasal suture could have been to avoid excluding the affected radiographs. This Nasion estimation was only applied for the determination of the Bjork sum and the Frankfort-mandibular-plane-angle according to Ricketts.

**Table 1:** Cephalometric landmarks used in this study

Landmark	Abbreviation	Description
Nasion	N	Most anterior point on the frontonasal suture
Sella	S	Midpoint of the Sella turcica
Porion	Po	The most cranial point of the upper margin of the external auditory meatus
Orbitale	Or	The most caudal point of the orbital margin
Gonion	Go	Most posterior inferior point on the mandibular angle. It is constructed by bisecting the angle formed by intersection of mandibular plane and a tangential line at the ramus of mandible
Menton	Me	Most caudal point on mandibular symphysis

**Table 2:** Cephalometric lines used in this study

Line	Abbreviation	Description
Frankfort horizontal plane	FHP	Connecting line between Or and Po
Nasion-Sella-line	NSL	Connecting line between the S and N
Mandibular plane	Go-Me	Connecting line between Go and Me

**Table 3:** Cephalometric angles used in this study

Angle	Abbreviation	Description
FHP-NSL-angle	FHP-NSL	The angle between the Nasion-Sella-line (NSL) and the Frankfort horizontal plane (FHP)
Facial horizontal plane	FHP-Burstone	The facial horizontal plane is tilted 7° clockwise to the Nasion-Sella-line (NSL)
NHP-discrepancy	NHP-discrepancy	The discrepancy of the Natural Head Position (NHP) from an ideal alignment of the head parallel to the floor using the formula:(β = the angle between the NSL and right image border)NHP-discrepancy < 0° = counter clockwise rotation of the headNHP-discrepancy > 0° = clockwise rotation of the headNHP-discrepancy = 0° = ideal position of the head (NHP parallel to the floor)
Sum of Bjork polygon angles	Bjork sum	The sum of the saddle angle (N-S-Ar), the articular angle (S-Ar-Go) and the gonial angle (Ar-Go-Gn)>= 400° = backward growth rotation of the mandible<= 392° = forward growth rotation of the mandible
Mandibular plane angle	MA	The angle between the FHP and the mandibular plane25° ± 5° = normal range>= 30° = high-angle patient<= 20° = low-angle patient
FHP-discrepancy	FHP-discrepancy	The angle between the Frankfort Horizontal Plane (FHP) and the vertical image border90° = correct head positioning<= 90° = backward head inclination>= 90° = forward head inclination

#### Bjork sum

The Bjork sum was calculated by summarizing the saddle angle (N-S-Ar), the articular angle (S-Ar-Go), and the gonial angle (Ar-Go-Gn). The following interpretation was employed: If the resulting angle was greater than 400°, the mandible demonstrated a clockwise growth rotation, whereas if the total was less than 392° the mandible demonstrated a counter clockwise growth rotation. Hence, resulting angles between 392°-400° indicated a neutral growth pattern of the mandible [9].

#### Frankfort-mandibular-plane-angle according to Ricketts

The Frankfort-mandibular-plane-angle according to Ricketts denotes the angle between the Frankfort Horizontal Plane (FHP) and the mandibular plane (Go-Gn). The following interpretation was utilized: Within the range of 25° ± 5° the angle was considered to be within normal range, whereas a high-angle, meaning vertically growing patient, had a mandibular plane angle of 30° or more and a low-angle, meaning horizontally growing patient, showed a value of 20° or less [11].

#### FHP-NSL-angle

The angle between the FHP and NSL (FHP-NSL-angle) was measured using the open-source image processing programme Fiji [8]. This angle demonstrates the inclination of the anterior cranial base relative to the Frankfort horizontal plane. Since NSL is assumed to correlate with the Natural Head Position (NHP), it was assessed to investigate whether patients subject to landmark masking had a larger discrepancy between their natural head position and the FHP used for positioning in the x-ray machine.

Daugaard-Jensen examined the relationship between these two planes in 1957 and noted an almost constant angle of 7° during growth [12], though it varies individually. A higher FHP-NSL-angle indicates a more vertical orientation of the cranial base, whereas a smaller FHP-NSL-angle indicates a more horizontal orientation of the cranial base [13].

#### FHP-discrepancy

Since patients were supposed to be oriented according to the Frankfort horizontal plane when the image was recorded, the angle between FHP and the vertical image border (right side) was also calculated to assess the accuracy of patient positioning. In case of correct head positioning, the FHP-discrepancy should amount to 90°. Smaller angles represent backward inclination of the head, whereas higher angles indicate forward inclination.

**Fig. 2:**
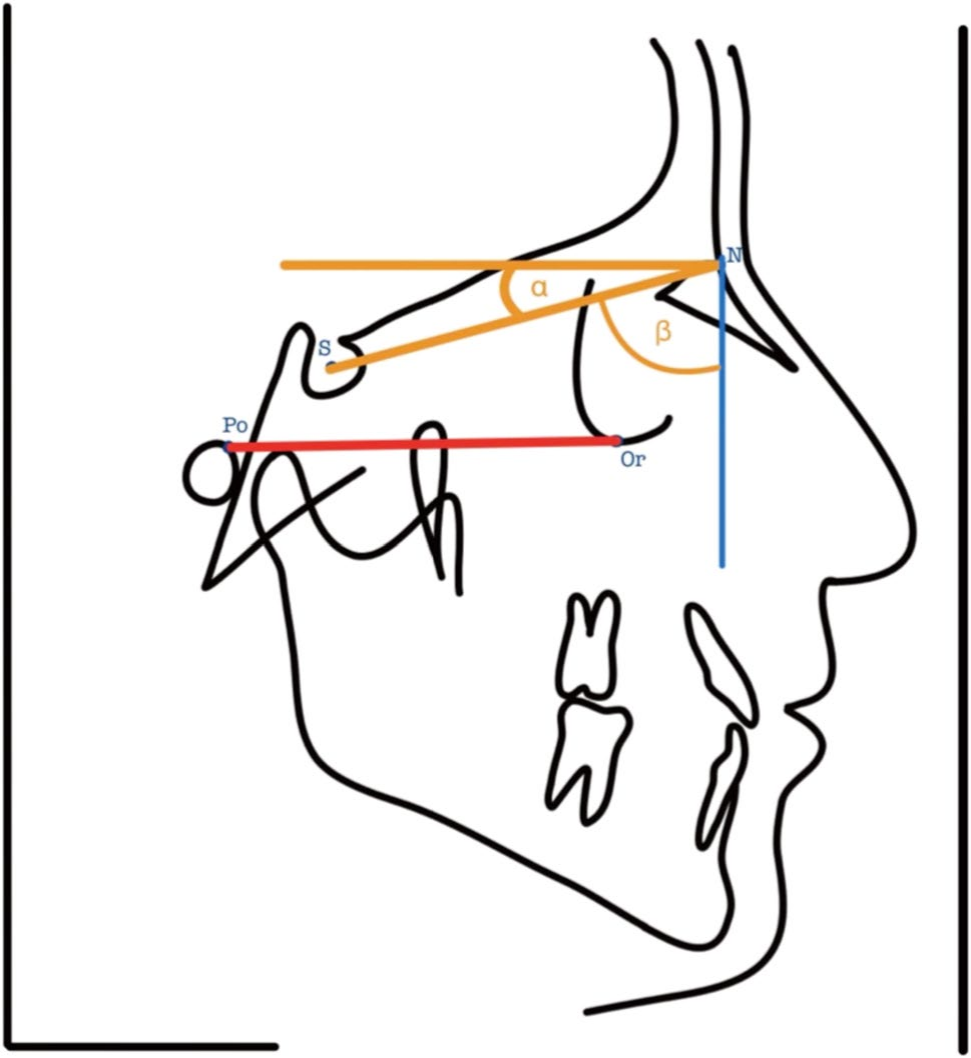
Assessment of parallelity of the facial horizontal plane to the patient’s “true” facial horizontal plane. α = 7° (angle between the facial horizontal plane and the NSL). β = 83° (angle between the NSL and right image border)

#### Natural head position (NHP-discrepancy)

According to Burstone, the facial horizontal plane is a line that passes through the *Nasion* and is inclined 7° clockwise relative to the Nasion-Sella-line (NSL) (Fig. 2, α angle) [14]. It represents the Natural Head Position (NHP), which has been suggested as an alternative to the FHP to align patients in the x-ray machine for taking a lateral cephalogram. This plane is not tied to facial reference points and reflects the self-balanced posture of a person looking straight ahead [15]. The facial horizontal plane would be directed orthogonal to the vertical image boundary in the lateral cephalogram in case that patients were positioned with respect to their NHP instead of the FHP.

The NHP and the FHP are distinct planes, even though they share a similar angular relationship of 7° with the Nasion-Sella-line (NSL). While the FHP is an anatomically defined reference plane, the NHP depicts the natural head posture of a patient when looking straight forward. The FHP-NSL-angle has to be determined individually whereas the angle between NSL and NHP is a theoretical standard value.

To assess to what extent the facial horizontal plane was indeed parallel to the floor during image capturing (or orthogonal to the vertical image border at the right side), an angle β was defined between the Nasion-Sella-line and the vertical image boundary (Fig. 2).

Therefore, in case of NHP being parallel to the floor, the angle β (between the vertical image boundary and NSL) would amount to 83°. To calculate the positioning discrepancy, the β angle (Fig. 2) was measured and the result was then subtracted with 83° and defined as *NHP-discrepancy* as it is representing the angular discrepancy of the facial horizontal plane to a line parallel to the floor. Hence, the following formula was used (assuming $\:\alpha\:=7^\circ\:)$:

$$\:NHP-discrepancy=\beta\:-\:83^\circ\:$$

Values of $\:\beta\:$ smaller than 83° therefore represented counter clockwise rotation of the head while $\:\beta\:$ larger than 83° represented a clockwise rotation.

Thus, in case of *NHP-discrepancy* of 0° ideal head positioning was assumed. Values of $\:NHP-discrepancy>$ 0° therefore represented a forward/clockwise inclination of the head, whereas values lower than 0° represented a counter clockwise rotation of the head.

### Data analysis

Statistical analysis was performed using the open-source software R [16]. Association of the skeletal growth pattern according to the Bjork sum and the *Rickett’s mandibular plane angle*, the *FHP-NSL-angle*,* the FHP-discrepancy* and the *NHP-discrepancy* with landmark masking was achieved by means of logistic regression. Masking of cranial reference points was defined as the dependent variable, i.e. 1 = masking and 0 = no masking. Results were found significant at *p* < 0.05.

## Results

### Collimation analysis

**Fig. 3:**
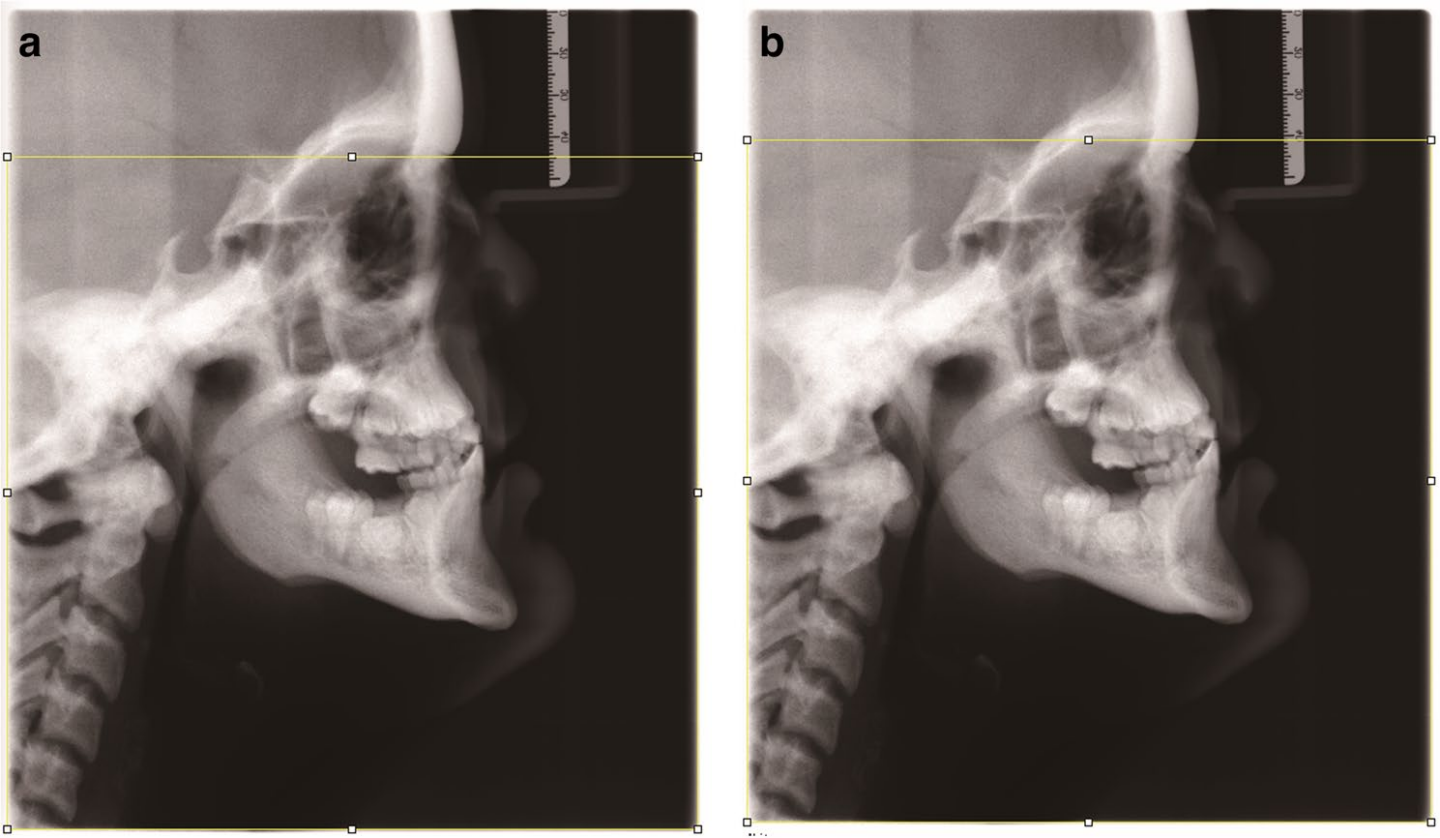
**a** Masking of Nasion after virtual collimation using the geometry provided by the manufacturer. **b **No masking of Nasion after virtual collimation of the upper 16.02% of the image height

*Sella* was visible on all radiographs (100%) implicating that no *Sella* masking was observed in any radiograph. *Nasion* was masked in 0.6% (*n* = 6) of the lateral cephalograms (Fig. 3a).

In detail, it was masked in 4 of the lateral cephalograms after virtual collimation (66.67%) and in 2 of the lateral cephalograms that were captured with cranial collimation (33,33%).

Of the six cephalograms with landmark masking, 50% were from patients with a vertical growth pattern according to the Bjork sum and the Frankfort-mandibular-plane-angle. The other 50% were taken from patients with a mesofacial growth pattern but who had also a backward head inclination.

No masking of *Nasion* would occur if only the upper 16.02% of the image height had been collimated (Fig. 3b).

### Association of landmark masking and growth pattern

**Fig. 4:**
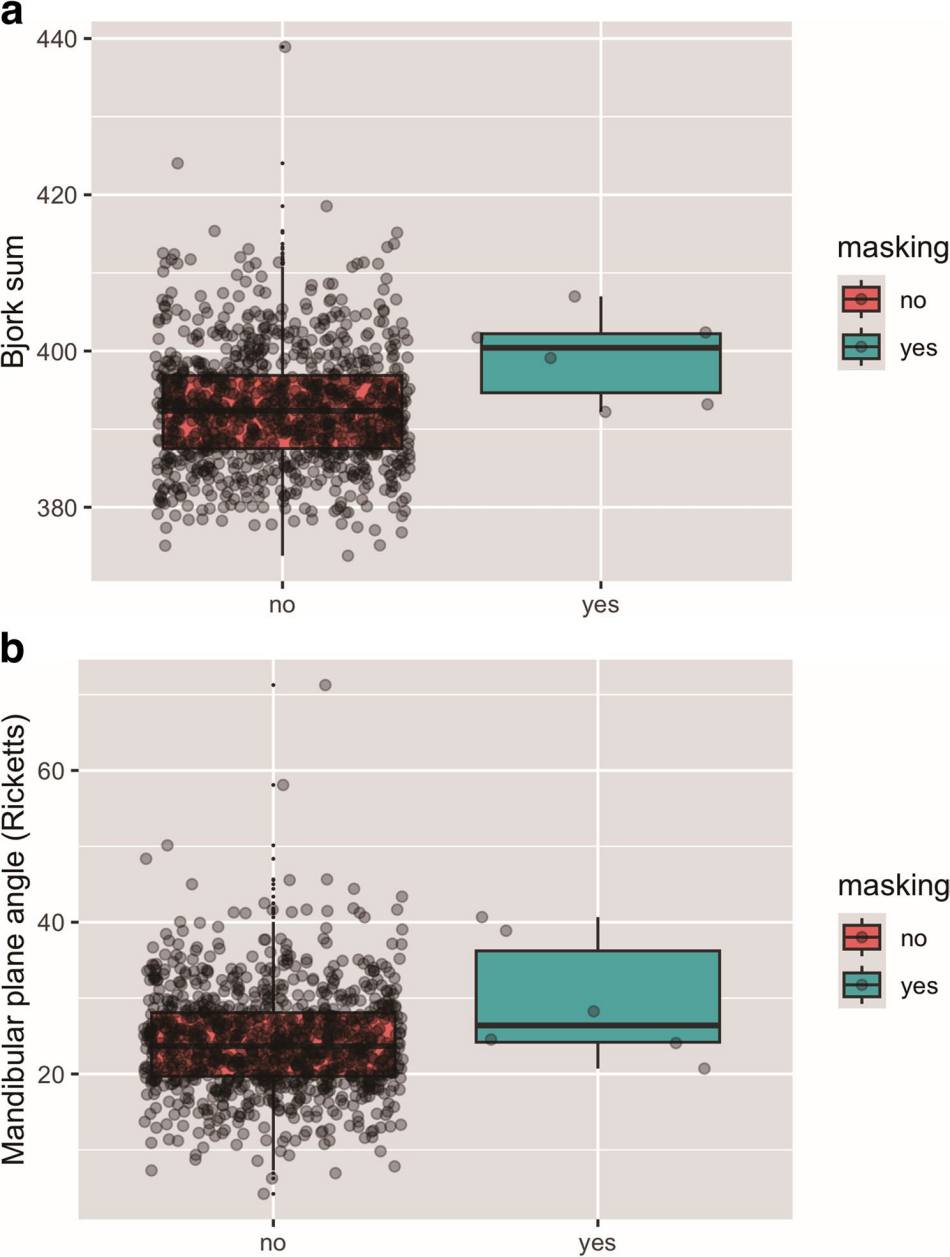
**a** Representation of Bjork sum for lateral cephalograms with/ without landmark masking. **b **Representation of mandibular plane-angle (Ricketts) for lateral cephalograms with/ without landmark masking

Based on the analysis of the Bjork sum, most patients exhibited a horizontal growth pattern (*n* = 473; 47.3%), followed by a mesofacial growth pattern (*n* = 370; 37%) and a vertical growth pattern (*n* = 157; 15.7%). The mean Bjork sum amounted to 392.77° ± 7.51°. In patients who did not exhibit landmark masking, it amounted to 392.70°±7.50° whereas in patients with masking it amounted to 399.26° ± 5.70°. Logistic regression confirmed significant association of growth pattern and landmark masking (*p* = 0.029) (Fig. 4a). This indicates that a more vertical growth pattern according to the Bjork sum is associated with an increased possibility of landmark masking.

Based on the analysis of the mandibular plane angle according to Ricketts, most patients showed a mesofacial growth pattern (*n* = 551; 55.1%), whereas the number of horizontally growing patients (*n* = 273; 27.3%) and vertically growing patients (*n* = 176; 17.6%) was lower.

Overall, patients had an average mandibular plane angle of 24.25°± 6.87°. In patients who did not exhibit masking, it amounted to 24.22°± 6.85° whereas in patients with masking it amounted to 29.52° ± 8.31°. Logistic regression showed a trend toward significance, but the association was not statistically significant (*p* = 0.051) (Fig. 4b).

The average FHP-NSL-angle on all lateral cephalograms amounted to 9.61° ± 3.02° demonstrating an overall more vertical orientation of the cranial base.

On the lateral cephalograms where landmark masking occurred, the average FHP-NSL-angle amounted to 9.42° ± 3.85°, whereby two physically collimated images were not taken into account because Nasion was masked.

**Fig. 5:**
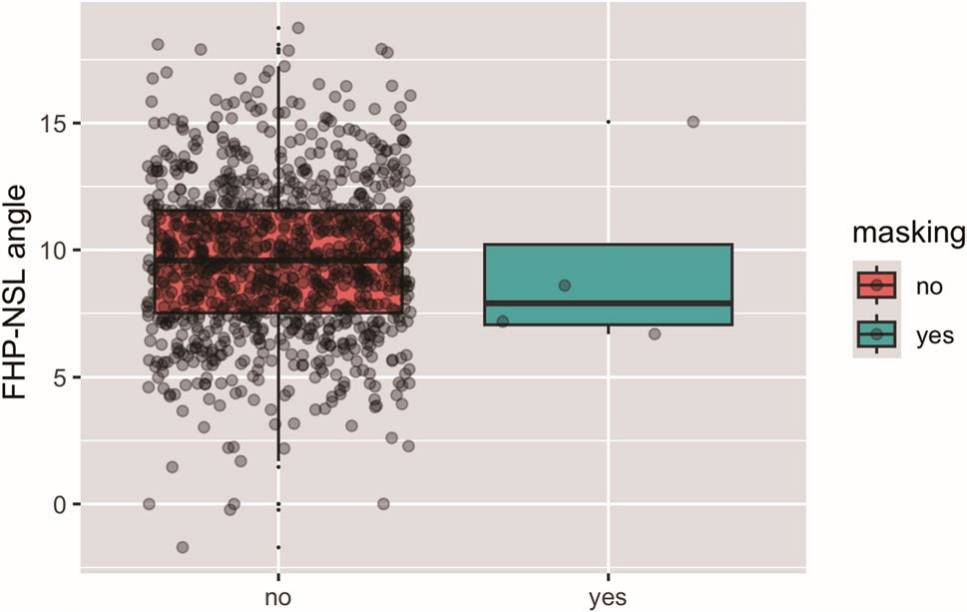
FHP-NSL angle in lateral cephalograms with and without landmark masking

The log-regression did not reveal any association of the inclination of the anterior cranial base relative to the FHP in lateral cephalograms with landmark masking (*p* = 0.880) (Fig. 5).

### Association of landmark masking and positioning

**Fig. 6:**
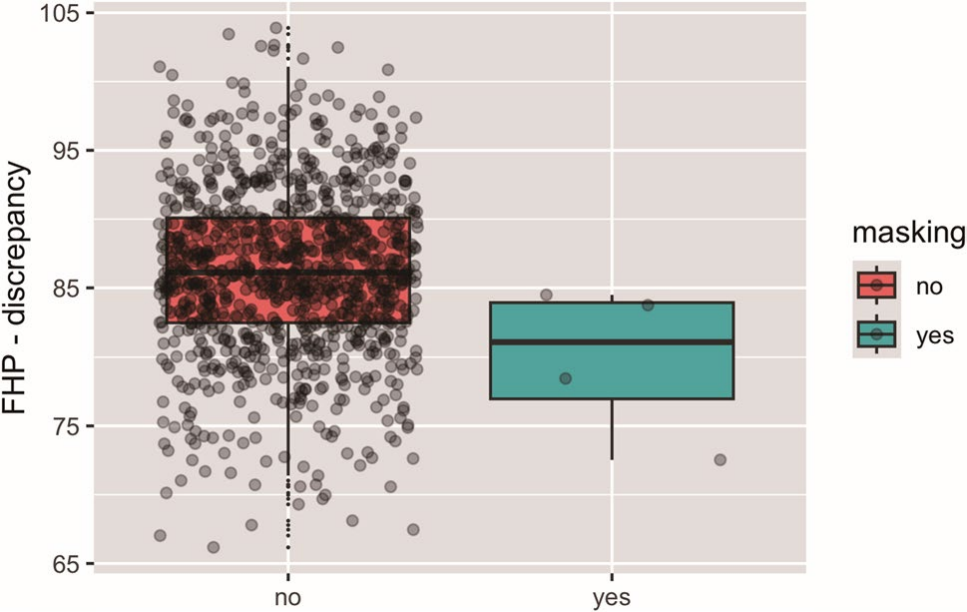
Association between landmark masking and positioning to the FHP. An angle of 90 degree would represent perfect positioning, whereas a lower amount represents backward inclination of the head

When assessing whether landmark masking was influenced by malpositioning in the x-ray machine when capturing the lateral cephalogram, log regression revealed a significant association between masking and FHP-discrepancy (*p* = 0.040) (Fig. 6), which indicated that landmark masking was significantly associated with malpositioning, i.e. counter clockwise inclination of the head.

**Fig. 7:**
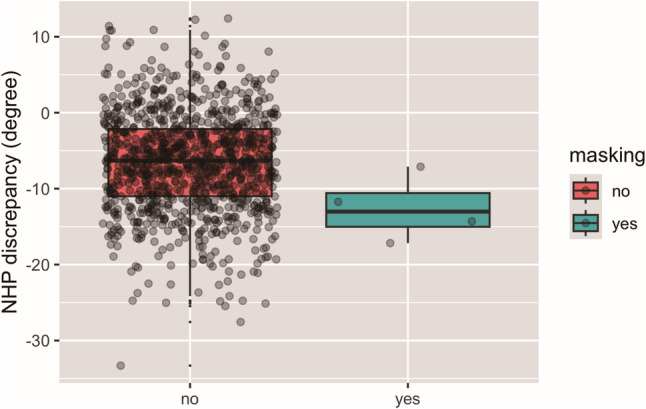
NHP-discrepancy in lateral cephalograms with and without landmark masking

Besides FHP-discrepancy, the NHP-discrepancy was also assessed and amounted on average to -6.57° ± 6.58°. On the cephalograms where landmark masking occurred (excluding the images with initial masking of Nasion), the NHP-discrepancy amounted to -12.58° ± 4.27° representing a more pronounced backward inclination of the head, whereby in the remaining patients, it amounted to -6.55 ° ± 6.57°. Despite this trend, the logistic regression failed to confirm correlation of the NHP-discrepancy with landmark masking (*p* = 0.064) (Fig. 7).

## Discussion

Growing patients are frequently subject to orthodontic treatment, and previous studies revealed that they receive a higher cumulative x-ray dose during adolescence compared to adolescents without orthodontic treatment [17]. Current regulations require reducing the effective dose “as much as reasonably achievable” (ALARA-principle) regardless of age [5]. The ALARA principle is sometimes also referred to as the ALADA-IP principle (“As low as diagnostically acceptable being indication-oriented and patient-specific”) [5]. This highlights that the justification of radiological images should address two aspects: the clinical relevance to capture a radiograph and the choice of imaging parameters including collimation that take the patients age and radiation sensitivity into consideration.

At the time being, there is no conclusive information regarding cranial collimation of lateral cephalograms in orthodontics, and it is unclear if collimation is associated with an increased risk of masking cranial landmarks. Landmark masking may necessitate repeating the radiological image and lead to a further increase of the overall radiation dose.

The present analysis revealed that by collimating the upper 18% of lateral cephalograms, *Nasion* was collimated in only 0.6% of patients. Although this is a very small number, it is necessary to consider the clinical implications. In clinical practice, the collimation of Nasion in 0.6% of 1000 patients means that 6 patients might have required re-imaging, potentially resulting in avoidable additional radiation exposure. From a radiation protection standpoint, even though the 0.6% incidence of landmark masking is relatively low, it still warrants investigation of the underlying causes and the development of strategies to reduce this risk to an absolute minimum.

When assessing the association of landmark masking and growth pattern, it was found that vertical growth pattern according to Bjork sum was significantly associated with landmark masking. The reason might be that longer faces are more closely positioned to the upper margin of the detector, which would increase the chance of Nasion masking. However, it may also be possible that patients with a vertical growth pattern are more frequently malpositioned with a counter clockwise orientation, and future studies are needed to assess if there is any correlation.

For the evaluation of the head inclination during image capturing, the NHP-discrepancy based on the facial horizontal plane proposed by Burstone and the discrepancy to the FHP were defined. Burstone’s facial horizontal plane and the FHP are important reference planes in the analysis of lateral cephalograms, since they offer valuable insights into the orientation and inclination of the head during image capturing. Schwarz proposed the FHP as a horizontal reference line in 1884 [18], which is still an internationally widely accepted reference plane because of its clinical practicability, and most commonly utilized as reference plane when recording lateral cephalograms in Germany.

However, due to the limitations of the FHP other authors such as Moorrees et al. [19] or Viazis [20] favoured the NHP for the ease of image taking as it is based on the natural position of the patient’s head. During image capturing, the patient needs to stand upright and look straight ahead into a mirror into their own eyes. The cephalostat is positioned in the external auditory canal on both sides of the patient for the prevention of head rotation. In 1959 Burstone et al. [14, 21] proposed that the facial horizontal plane is inclined 7° clockwise to the Nasion-Sella-line (NSL). This angle was determined based on studies that analyzed the relationship between the NSL and the NHP [15]. Burstone’s facial horizontal plane therefore represents a standardized horizontal reference plane in cephalometric analysis. Potential variations in the inclination of the head and their impact could be identified by including both the NHP-discrepancy and the discrepancy to the FHP. While the FHP-discrepancy revealed a significant association of head inclination with landmark masking, the NHP-discrepancy slightly failed to reveal significant association. The identification of the FHP may be susceptible to variability due to landmark identification errors, whereas the use Burstone’s facial horizontal plane is not tied to fixed anatomical landmarks, but it is still prone to variability due to individual malpositioning of the head during visual alignment by the patient. For the present study, Nasion was masked in two radiographs with physical collimation, and no proportional sample size reduction was performed in the images without masking.

Furthermore, by measuring the angle between the FHP and the NSL, information about the inclination of the anterior cranial base relative to the FHP could be gathered. The FHP serves as a reference for the actual horizontal plane of the patient´s head [22–27]. Our patient clientele exhibited a more vertical orientation of the cranial base since our analysis on average revealed higher values than the recommended 7° according to Daugaard-Jensen. Overall, this offers an understanding of the importance of the growth pattern or inclination of the anterior cranial base on landmark masking. However, larger or smaller angles than the recommended value can be observed due to variations in the patient sample, such as sex, age, ethnicity or genetics.

There were some limitations related to the present study:

First of all, this retrospective study was conducted at a single center, which might lead to regional selection bias (patient of similar ethnicity, lateral cephalograms recorded for patients within a typical treatment age). This may limit the applicability of the findings to other clinical contexts or patient populations. Secondly, locating the landmarks Porion (Po) and Orbitale (Or) for determination of the FHP is not as reliable as marking *Sella* and *Nasion* in lateral cephalograms [28, 29]. Third, the number of subjects showing landmark masking was very low, and as specified already, statistical analysis was sometimes impeded by the exclusion of patients with physical Nasion masking. While the low incidence of landmark masking is reassuring from a clinical perspective, it also limits the statistical power to draw definitive conclusions regarding its association with skeletal growth pattern or head positioning. It has to be noted that no sample size calculation was possible since there is a scarcity of studies investigation the visibility of landmarks following collimation. Only a technical note could be identified testing the eligibility of a specific collimator covering the entire brain, which reduced the entire radiation dose by one third [30]. Nevertheless, our analysis did reveal statistically significant associations between landmark masking and both vertical growth pattern and FHP-discrepancy. However, the overlap between the ‘masking’ and ‘non-masking’ groups in the box plots indicates that these factors alone may not fully explain the phenomenon.

Furthermore, additional variables—such as patient misalignment due to operator inexperience, inadequate instructions during positioning, or patient movement and distraction during imaging—may also contribute to unintended landmark masking. Since these variables were not measured or controlled in the present study, they might have acted as a source of possible confounding.

Further studies involving a higher number of subjects exhibiting landmark masking are needed to elucidate if patients with certain external features, such as e.g., long face, that usually goes hand in hand with a vertical growth pattern, should be examined without cranial collimation, or whether caution that correct positioning is achieved is sufficient.

It has to be noted that treatment effects or growth over time can be studied by superimposing consecutive cephalograms. The gold standard is the structural method according to Bjork [31–33]. This is deliberately not based on individual points, but on structures at the base of the skull. Some of these lie cranial to the SN line. Thus, collimation may also impede the superimposition and thus the analysis of therapeutic effects. An incision line for collimation with a certain distance cranial to the SN line, taking into account the required structures of the cranial base, could be elaborated in future investigations.

Other limitations of the current study include that cephalograms were not taken prospectively, and manual collimation had to be applied to the majority of images. Additionally, only sagittal deviations in the head position were assessed, whereby transversal positioning errors could not be detected. The interpretation of the head position assumes a regular structure of the head, which may not be present in each patient. Finally, it has to be noted that no restrictions were made regarding the inclusion of radiographs.

## Conclusion

In conclusion, using the collimation geometry available in the commercial x-ray machine, Nasion was the only landmark that was masked in 0.6% of patients, and occurred only in patients with a vertical growth pattern and/or a backward head position. To minimize the risk of landmark masking, correct head positioning is advised. By only collimating the upper 16.02% of the image height of the lateral cephalogram, no landmark masking would have occurred in the present study, and future studies should evaluate whether a reduced collimation geometry improves diagnostic safety without compromising radiation protection.

## Supplementary Information

Supplementary Material 1

## Data Availability

The data of this article cannot be shared due to data protection reasons.
